# SLC25A1-associated prognostic signature predicts poor survival in acute myeloid leukemia patients

**DOI:** 10.3389/fgene.2022.1081262

**Published:** 2023-01-06

**Authors:** Fangshu Liu, Suqi Deng, Yue Li, Juan Du, Hui Zeng

**Affiliations:** Department of Hematology, The First Affiliated Hospital of Jinan University, Guangzhou, China

**Keywords:** acute myeloid leukemia, SLC25A1, metabolism, prognosis, target therapy

## Abstract

**Background:** Acute myeloid leukemia (AML) is a heterogeneous malignant disease. *SLC25A1*, the gene encoding mitochondrial carrier subfamily of solute carrier proteins, was reported to be overexpressed in certain solid tumors. However, its expression and value as prognostic marker has not been assessed in AML.

**Methods:** We retrieved RNA profile and corresponding clinical data of AML patients from the Beat AML, TCGA, and TARGET databases (TARGET_AML). Patients in the TCGA cohort were well-grouped into two group based on *SLC25A1* and differentially expressed genes were determined between the *SLC25A1* high and low group. The expression of *SLC25A1* was validated with clinical samples. The survival and apoptosis of two AML cell lines were analyzed with *SLC25A1* inhibitor (CTPI-2) treatment. Cox and the least absolute shrinkage and selection operator (LASSO) regression analyses were applied to Beat AML database to identify *SLC25A1*-associated genes for the construction of a prognostic risk-scoring model. Survival analysis was performed by Kaplan-Meier and receiver operator characteristic curves.

**Results:** Our analysis revealed that high expressed level of *SLC25A1* in AML patients correlates with unfavorable prognosis. Moreover, *SLC25A1* expression was positively associated with metabolism activity. We further demonstrated that the inhibition of *SLC25A1* could inhibit the proliferation and increase the apoptosis of AML cells. In addition, a panel of *SLC25A1*-associated genes, was identified to construct a prognostic risk-scoring model. This *SLC25A1*-associated prognostic signature (SPS) is an independent risk factor with high area under curve (AUC) values of receiver operating characteristic (ROC) curves. A high SPS in leukemia patients is associated with poor survival. A Prognostic nomogram including the SPS and other clinical parameters, was constructed and its predictive efficiency was confirmed.

**Conclusion:** We have successfully established a SPS prognostic model that predict outcome and risk stratification in AML. This risk model can be used as an independent biomarker to assess prognosis of AML.

## Introduction

Acute myeloid leukemia (AML) is a malignant clonal disease, characterized by massive proliferation of abnormal blasts and naive cells and inhibition of normal hematopoiesis (Khwaja et al., 2016). AML is the most common type of acute leukemia in adults (Newell and Cook, 2021). This type of cancer usually progressed quickly if not treated (Stanchina et al., 2020). Approximately 20240 children and adolescents in the United States develop acute myeloid leukemia in 2021. Despite advancements in the therapy of AML, the treatment effect remains unsatisfactory (Pulte et al., 2020). The relative 5-year survival rate of AML is merely 29.3% and is associated with a poor prognosis and a high relapse rate, requesting better predictive model for risk stratification and precision medicine. Increasing attention has been paid on the dysregulated metabolism underlying the differences between AML and normal compartments (Ye et al., 2016; Jones et al., 2019; van Gastel et al., 2020). Drugs targeting key regulatory genes in metabolism, Enasidenib and Ivosidenib, isocitrate dehydrogenase (IDH) inhibitors as a case in point, have been developed to treat AML more effectively than conventional regimen (DiNardo et al., 2018; Stein et al., 2019). Therefore, it is necessary to select appropriate metabolic reference genes for disease management, personalized therapy, diagnosis and prognosis of AML patients.

Solute carrier (SLC) family genes are strongly correlated with metabolism. SLC transporters is a family of more than 300 membrane-bound proteins that maintain the integrity of cellular membranes and transport a variety of different substrates including nutrients and xenobiotics (Lin et al., 2015; Rives et al., 2017). The mitochondrial carrier family is the largest solute transporter family in humans (Ruprecht and Kunji, 2020). The mitochondrial citrate transporter gene, *SLC25A1*, belongs to mitochondrial carrier family SLC25 and maps on chromosome 22q11.21 (Nota et al., 2013). Citric acid transporter regulates citric acid transport bidirectionally between the mitochondria and cytoplasm (Kang et al., 2021). Citrate is one of the intermediates in the tricarboxylic acid cycle (TCA cycle) accompanied by NADPH generation. Therefore, it promotes tumor growth and survival by affecting tumor metabolism. *SLC25A1* plays an essential role in the initiation and progression of cancer. The overexpression of *SLC25A1* promotes tumor growth and survival, such as colorectal cancer and non-small cell lung cancer (Fernandez et al., 2018; Yang et al., 2021). However, the potential prognostic value of *SLC25A1* expression in AML remains unclear.

In this study, high expression level of *SLC25A1* indicated a poor prognosis. We constructed a prognostic risk model based on *SLC25A1* and demonstrated that our prognostic risk model is efficient in predicting the prognosis of AML patients. Different cohorts were used to validate this prognostic model by survival analysis, independent prognostic analysis, and receiver operating characteristic (ROC) curve analysis. Moreover, we refined the 2017 European Leukemia Net recommendations for the genetic risk stratification of AML (ELN 2017) classification by adding *SLC25A1*-associated prognostic signature (Döhner et al., 2017). In addition, a clinical model was constructed that consisted of clinical predictors only (age, risk score, ELN 2017), which has a higher prediction accuracy than ELN2017 and provides a potential theoretical basis for clinical application.

## Materials and methods

### Data sources

The RNA-seq and matched clinical data of the Beat AML (n = 341) were downloaded from the cBio Cancer Genomics Portal (https://www.cbioportal.org/). The transcriptomic and clinical data of AML samples (n = 151) from The Cancer Genome Atlas (TCGA) database, the normal control obtained from GTEx Whole Blood (n = 337). TARGET_AML datasets (log_2_ (fpkm+1), n = 132) were collected from the University of California Santa Cruz database (UCSC Xena, https://xenabrowser.net/datapages/). RNA count data were transformed into log_2_ (TPM+1). The normalized microarray data of GSE63270 (n = 104), GSE13159 (n = 579) and GSE71014 (n = 208) were downloaded from the Gene Expression Omnibus (GEO) official website (http://www.ncbi.nlm.nih.gov/geo).

### Patients and ethics

The BM samples were obtained from 12 AML patients and 5 healthy donors at The First Affiliated Hospital of Jinan University from March 2019 to April 2022. The French–American–British (FAB) classification of AML patients was determined according to the 2016 World Health Organization (WHO) criteria. This study was approved by the Ethics Committee of The First Affiliated Hospital of Jinan University in accordance with the principles of the Declaration of Helsinki. All participants offered their written informed consents.

### LinkedOmics database

The LinkedOmics database (http://www.linkedomics.org/login.php) is acknowledged as a web portal that analyses multi-omics data from TCGA datasets (Vasaikar et al., 2018). We used LinkedOmics to study the *SLC25A1*-associated genes.

### GO and KEGG of *SLC25A1*-associated prognostic signature

R packages “limma” (https://bioconductor.org/packages/release/bioc/html/limma.html) was used to identify the differentially expressed genes (DEGs) between low-risk and high-risk groups within Beat AML cohort at adjusted *p*-value <.05 and |log_2_Foldchange (FC)| ≥2. Then the pathway enrichment analysis, including the Gene Ontology (GO) for “Biological Processes (BP)” category and the Kyoto Encyclopedia of Genes and Genomes (KEGG) pathway enrichment analysis were analyzed using R package “ClusterProfiler” (version 4.2.2).

### Construction and validation of the a *SLC25A1*-associated gene prognostic risk-scoring model

First, the “limma” package was used to identify the differentially expressed genes (DEGs) between AML and healthy donors in the TCGA dataset at *p*-value <.05 and |log_2_FC|≥1. A total of 4297 DEGs were intersected with *SLC25A1*-related genes obtained from Linkedomics with a criteria of the Spearman coefficient |R|≥.3 and *p*-value <.05. Next, we conducted the least absolute shrinkage and selection operator (LASSO) Cox regression model to screen prognostic signature in the Beat AML training set based on the *SLC25A1*-related intersection DEGs. The optimal lambda (λ) was selected by cross-validation error curve through the minimum 10-fold cross validation within the training set. Finally, a multivariate Cox regression analysis was conducted to establish a multi-genes classifier for predicting the overall survival (OS) of AML patients, and the risk score was calculated as the following formula:

$$Riskscore=\overset{n}{\underset{i=1}{\sum }}\left(Coefi\times x_{i}\right)$$

Coefi, coefficient. x_i_, z-score-transformed relative expression value of each gene.

After constructing the risk model, AML patients from the Beat AML dataset were divided into high- and low-risk groups using the optimal cutoff value computed by the “surv_cutpoint” function of R package “survminer”. The survival outcome between these groups was analyzed by Kaplan-Meier, and 1-, 3-, and 5-year receiver analysis were performed by the “time-ROC” R package. To examine the predictive accuracy of the model, two more AML cohorts from the TCGA and TARGET databases (TARGET_AML) were used for validation using the same risk score algorithm followed by risk subgrouping, survival analysis and ROC curves. Optimal cutoff value were determined by the ‘surv_cutpoint’ function of R package “survminer”.

### Prognostic independence of the risk-scoring model

We extracted clinical information from Beat AML database, and the univariate and multivariate Cox regression analysis were used to identify the independent prognostic factors.

### Construction of the predictive nomogram

We generated a nomogram to predict the 1-year, 2-year and 3-year overall survival of AML patients from the Beat AML cohort using “rms” R package. Calibration curves were applied to assess the predictive accuracy with self-validation done every 80 patients itinerantly for better stability.

## Cell count kit (CCK8) assay

AML cell with different pretreatments were plated (1 × 10^4^ cells/well) into 96-well plates and cultured in growth medium at 37 °C for 48 h. 10 µL Cell Counting Kit-8 (CCK-8, MedChemExpress, Shanghai, China) reagent was added into each well for 1–2 h incubation. Absorbance was determined at 450 nm. The viability of cells was calculated as following: Viability = (OD_test group-_OD_blank group_)/(OD_control group-_OD_blank group_) × 100%, and IC50 (half maximal inhibitory concentration) was calculated from the dose–response curves. Each experiment was performed in triplicate.

### Apoptosis analysis

Cells were seeded into 12-well plates at a density of 2 × 10^5^cells/mL with or without *SLC25A1*-specific inhibitor CTPI-2 for 48 h. Cells were harvested, washed with PBS and staining was performed with the Annexin-V/PI Apoptosis Detection Kit (BD Pharmingen, 556547, United States) following the manual. After incubation for 15 min, the samples were analyzed for apoptotic proportions using a BD FACSCanto II flow cytometer (BD Biosciences, Bedford, MA). Apoptotic percentages of the treated cells were analyzed and plotted with FlowJo software (Version10.4).

### Quantitative real-time PCR

Total RNA of AML patients and health donor was extracted using TRIzol (Life Technologies). Evo M-MLV RT Premix for qPCR (AG, AG11706, China) was used to synthesize cDNA. Quantification of transcripts was performed *via* the SYBR^®^ Green (Accurate Biology) according to the manufacturer’s instructions. Real-time PCR results are presented as the mean of three independent experiments normalized to β-actin internal control gene expression. The sequences of PCR primers as *SLC25A1* forward: 5′-CCC​CAT​GGA​GAC​CAT​CAA​G-3′, reverse: 5′ -CCT​GGT​ACG​TCC​CCT​TCA​G-3′, Relative gene expression was calculated using the 2^−ΔΔCT^ method.

### RNA sequencing

Lineage depletion was performed with biotin-lineage cocktail (CD2, CD3, CD4, CD8a, CD10, CD19, CD20, CD235a) and anti-biotin microbeads (Miltenyi Biotec, Germany). RNA was extracted from these 12 bone marrow samples of AML patients using TRIzol reagent. Sequencing library construction and sequencing was performed by Novel Bioinformatics (Shanghai, China). A total of 12 samples were sequenced using the Illumina NovaSeq 6000 platform.

### Statistical analysis

All statistical analysis were performed in R (version 4.0.3). Survival analysis were performed the “survival” and “survminer” packages. The “survivalROC” package in R was used to calculate area under the curve (AUC) values and construct ROC curves. The “rms” R package constructed nomogram and the consistency between observed and predicted risk was analyzed using Harrell’s C-statistic. Univariate and multivariate Cox regression analysis were performed using SPSS software version 26.0. All tests were two-sided, and *p*-value <.05 was considered as statistically significant.

## Results

### *SLC25A1* expression was elevated and correlated with worse survival in AML patients

**FIGURE 1:**
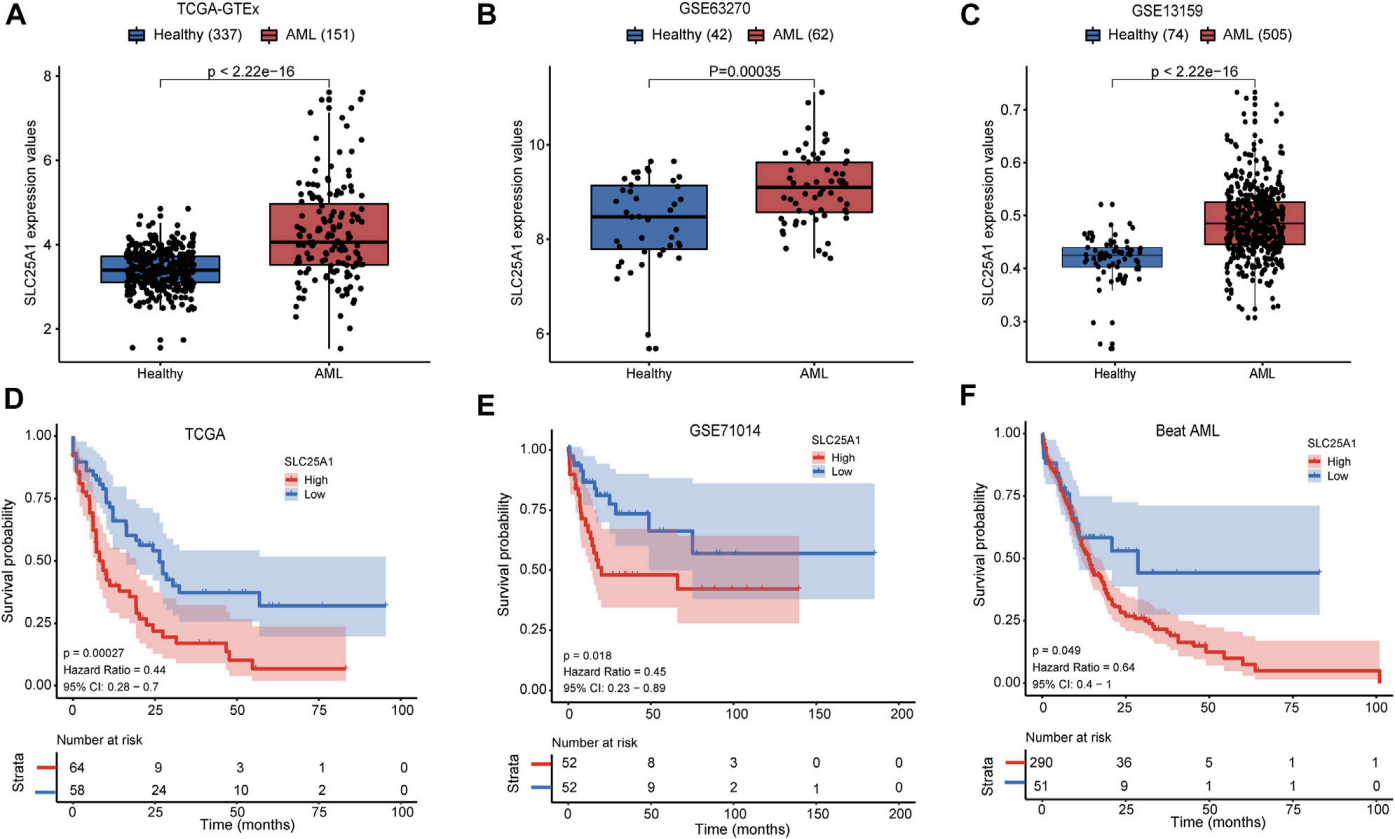
*SLC25A1* expression patterns and Kaplan-Meier survival analysis. (**A–C**) The *SLC25A1* mRNA expression level between AML patients and healthy donors in the TCGA-GTEx cohort, GSE63270 cohort and GSE13159 cohort, respectively. **(D–F)** Kaplan-Meier survival analysis of *SLC25A1* mRNA expression grouping by optimal cutoff value using “survminer” package in the TCGA, GSE71014 and Beat AML datasets.

To compare the expression level of *SLC25A1* in AML patients and healthy donors, we extracted the *SLC25A1* expression values of these groups from the TCGA x GTEx datasets, GSE63270 and GSE13159 respectively. The *p*-values calculated from Wilcoxon test are shown. We observed that *SLC25A1* had a significantly higher expression level in AML patients compared to healthy donors (*p* < .01) (Figures 1A–C). Next, we examined whether elevated expression level of *SLC25A1* was associated with poor prognosis. We set the value as cut off by the “survminer” package and separated each dataset into two groups, *SLC25A1* high and low expression. Firstly, we analyzed the correlation of gene expression profiles of *SLC25A1* and overall survival (OS) from TCGA dataset. AML patients with high expression level of *SLC25A1* gene had shorter overall survival (OS), indicating a poor prognosis than those with low expression level of *SLC25A1* gene (*p* = .00027) (Figure 1D). Additionally, similar results could be obtained in both GSE71014 and Beat AML datasets (*p* = .018, *p* = .049, respectively) (Figures 1E, F). Collectively, our analysis results indicated that *SLC25A1* expression level was elevated in AML patients and this expression signature significantly associated with poor OS.

### *SLC25A1* is positively correlated with oxidative phosphorylation in AML

**FIGURE 2:**
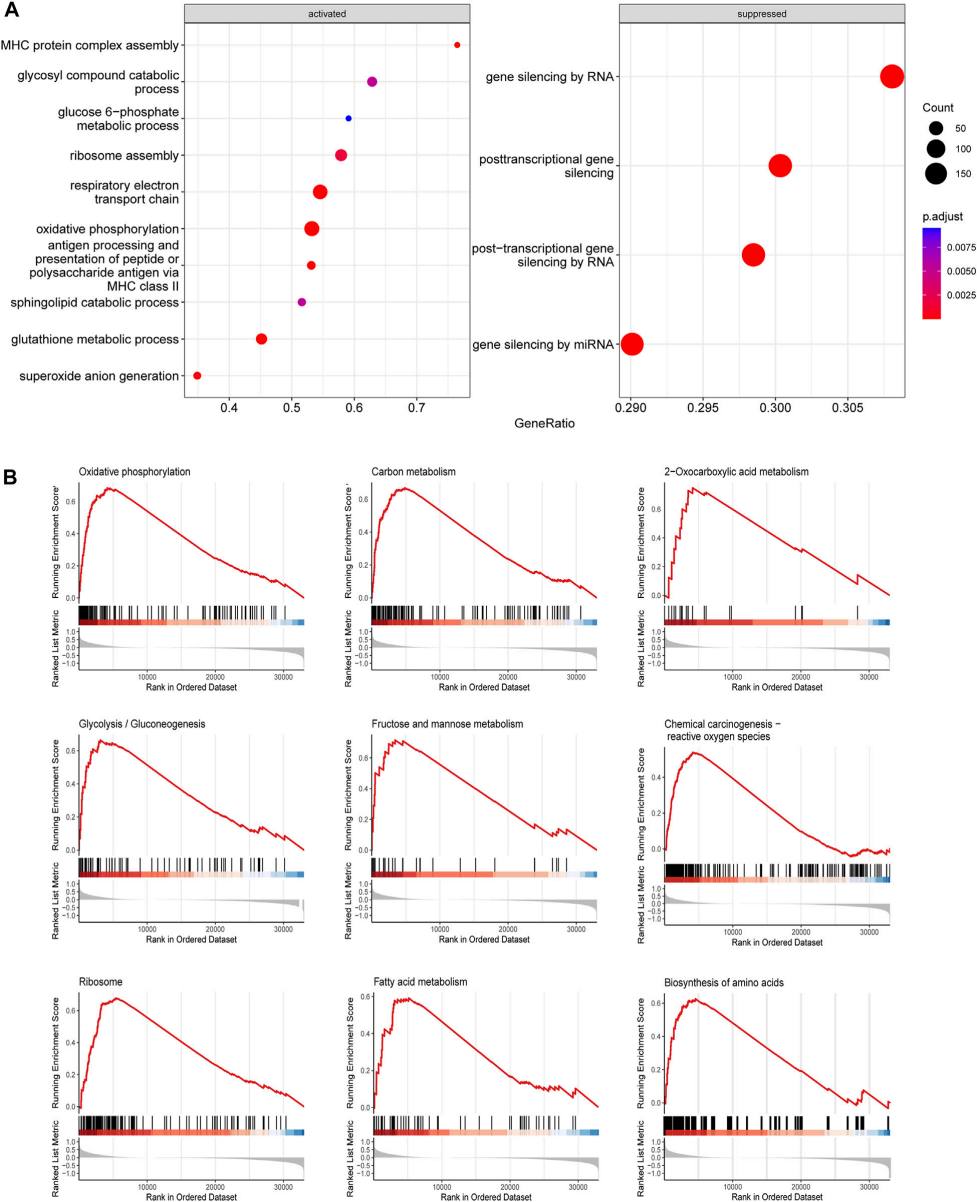
Gene ontology (GO) and Gene Set Enrichment Analysis (GSEA) associated with *SLC25A1* expression **(A)**. Top 10 GO terms of BP category enriched in *SLC25A1* high-expression groups compared with low-expression **(B)**. KEGG functional enrichment analysis of the same genelist in **(A)**.

The functions of *SLC25A1* in AML is poorly understood. To gain insights into the potential molecular mechanisms underlying function of *SLC25A1* in AML, we performed transcriptomic analysis of TCGA database between *SCL25A1* high and low subgroups. AML patients were separated into these two subgroups according to median value of *SCL25A1*. We obtained 901 upregulated genes and 2175 downregulated genes between *SLC25A1* ^*hi*^ and *SLC25A1* ^*low*^ subgroup (Supplementary Table 1). GO analysis of these differentially expressed genes (DEGs) showed activation of glucose metabolism-related pathways, respiratory electronic transport chain, oxidative phosphorylation and so on in *SLC25A1* ^*hi*^ subgroup (Figure 2A). To identify potential signaling pathways downstream of *SLC25A1*, we performed the Gene set enrichment analysis (GSEA) of DEGs. As shown in Figure 2B and Supplementary Table S2, metabolic pathways such as Oxidative phosphorylation, carbon metabolism, fatty acid metabolism, were remarkably enriched in *SLC25A1* ^*hi*^ subgroup (FDR <.05). Taken together, these results suggest that *SLC25A1* is positively correlated with oxidative phosphorylation in AML.

### Inhibition of *SLC25A1* suppresses the growth and induces apoptosis in AML cells

**FIGURE 3:**
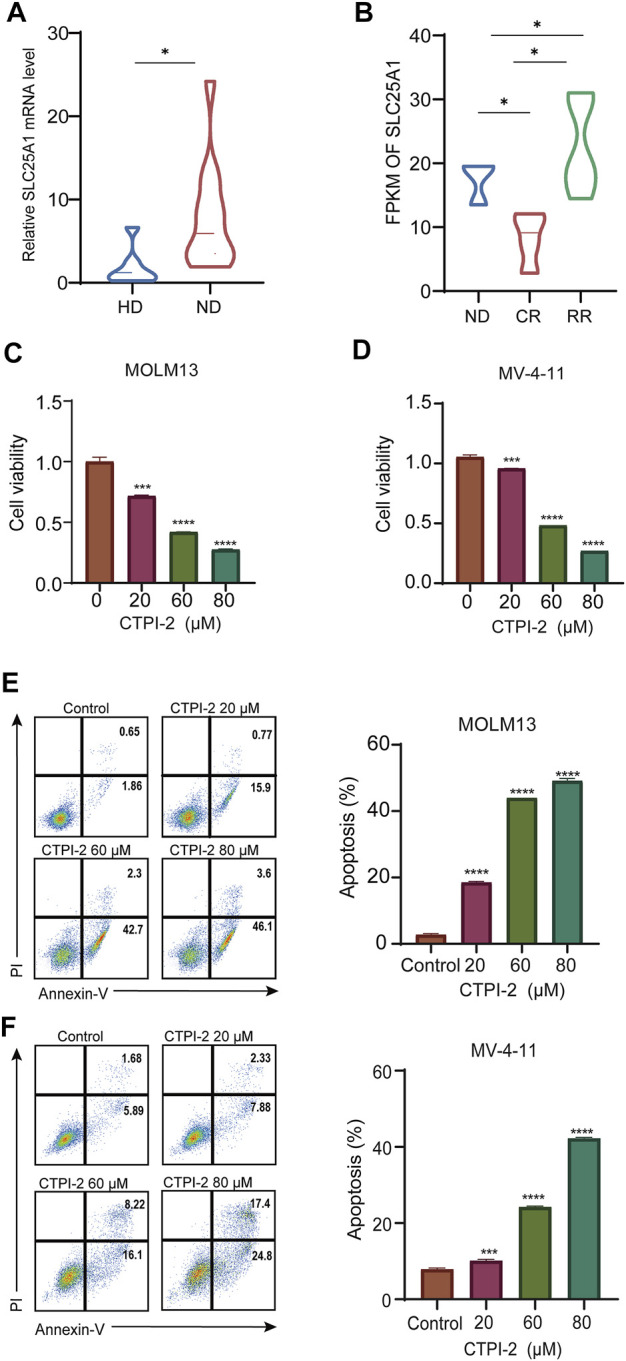
Inhibition of *SLC25A1* suppressed the growth and induced apoptosis in AML cell lines **(A)**. *SLC25A1* mRNA expression of AML patients and healthy donors from the First Affiliated Hospital of Jinan University **(B)**. Expression pattern of *SLC25A1* among various disease status in AML **(C,D)**. CCK-8 assay was used to investigate the IC50 of MOLM13 and MV-4–11 after treating with CTPI-2, a *SLC25A1* antagonist for 48 h **(E,F)**. Annexin-V/PI apoptosis analysis of MOLM13, MV-4–11 treated with CTPI-2 or DMSO by flow cytometry. Cells were treated with CTPI-2 for 48 h at indicated concentrations. *p < .05, **p < .01, ***p < .001.

To further validate *SLC25A1* expression, we collected bone marrow samples from 12 AML patients for qPCR. Our results showed that *SLC25A1* expression was significantly higher in newly diagnosis (ND) AML group than the healthy donor (HD) (*p* < .05) (Figure 3A). The expression of *SLC25A1* was also further validated with another lymphocyte-depleted transcription dataset of AML samples generated in Department of Hematology of the First Affiliated Hospital of Jinan University. We found FPKM of both ND patients and relapsed/refractory (RR) patients were significantly higher than that of complete remission patients (CR) (*p* < .05) (Figure 3B). Next, we tested whether inhibition of *SLC25A1* with its inhibitor CTPI-2 would suppress growth of AML cells. CCK8 assay results indicated that cell viability of AML cells was significantly reduced with CTPI-2 treatment for all concentrations (20 μM, 60 μM, and 80 μM) in MV-4-11 and MOLM13 cells (Figures 3C, D). Consistently, apoptosis was effectively induced in MOLM13 cells, MV-4-11 (Figures 3E, F). Taken together, we demonstrated that the upregulation of *SLC25A1* in AML bone marrow samples was validated and inhibition of *SLC25A1* could efficiently suppress the growth and induce apoptosis of AML cells. Therefore, *SLC25A1* may be an ideal candidate for the construction of prognostic model in AML.

### Construction and validation of prognostic model based on *SLC25A1*

**FIGURE 4:**
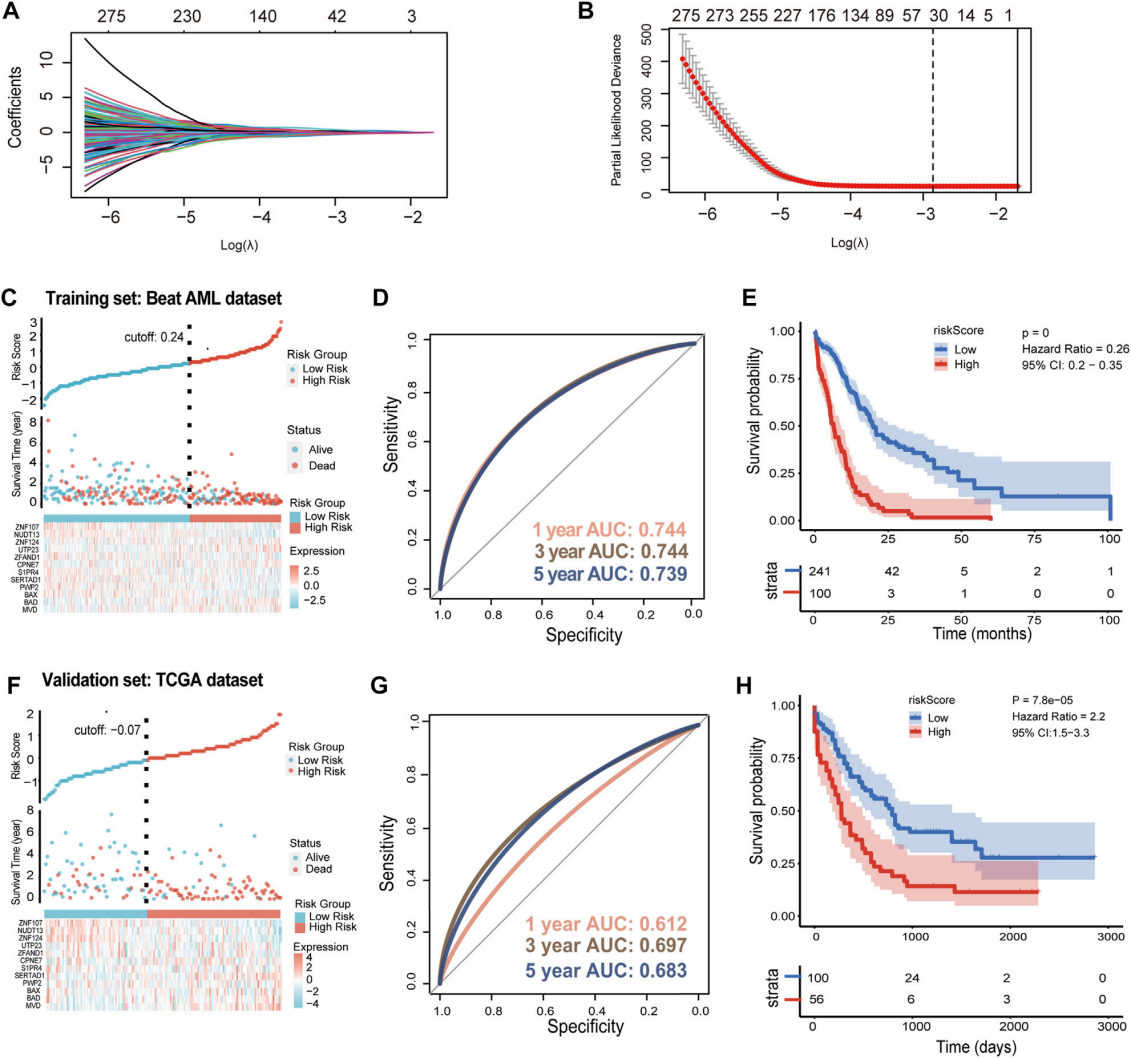
Prognostic implications of *SLC25A1*-associated prognostic signature in AML dataset **(A)**. LASSO coefficient profiles for the *SLC25A1*-related differentially expressed genes **(B)**. 32 candidate genes were screened out by LASSO regression with tenfold cross-validation using minimum lambda value **(C)**. Establishment of the *SLC25A1*-associated prognostic signature (SPS) model dividing patients into high and low risk groups in the training set **(D)**. The 1-, 3-, and 5-year ROC curves of risk score of the Beat AML training set **(E)**. Kaplan-Meier curves showing percentages of surviving patients of two risk groups **(F)**. Establishment of the *SLC25A1*-associated prognostic signature (SPS) model dividing patients into high and low risk groups in the TCGA validation set **(G,H)**. The 1-, 3-, and 5-year ROC curves of risk score and Kaplan-Meier curves in TCGA validation set. *p*-value was calculated using the log-rank test.

To construct the prognostic model, we firstly identify 4297 DEGs between AML patients and healthy people from TCGA-GTEx dataset (Supplementary Table S3). In addition, a total of 3735 *SLC25A1*-associated genes were obtained using LinkedOmics, including both positive and negative associations (Supplementary Table S4). These differential genes of TCGA-GTEx dataset and *SLC25A1*-associated genes were used to construct a Venn diagram for intersection, and 787 DEGs in common were obtained (Supplementary Figure S1). To further identify *SLC25A1*-associated genes for model construction, LASSO Cox regression analysis was performed on these 787 genes to find out the prognostic genes in the training set. The LASSO algorithm and stepwise multivariate Cox regression analysis were applied. At the minimum of λ value (λ = .05692), thirty-two prognostic signature genes were selected from 787 genes (Figure 4A), and their LASSO coefficient curves were shown in Figure 4B. Eventually, twelve prognostic signature genes related *SLC25A1* were selected after multivariate regression analysis. Mevalonate Diphosphate Decarboxylase (*MVD*), BCL2 Associated Agonist Of Cell Death (*BAD*),BCL2 Associated X (*BAX*), PWP2 Small Subunit Processome Component (*PWP2*), SERTA Domain Containing 1 (*SERTAD1*), Sphingosine-1-Phosphate Receptor 4 (*S1PR4*), Copine 7 (*CPNE7*), Zinc Finger AN1-Type Containing 1 (*ZFAND1*), UTP23 Small Subunit Processome Component (UTP23), Zinc Finger Protein 124 (*ZNF124*), Nudix Hydrolase 13 (*NUDT13*), Zinc Finger Protein 107 (*ZNF107*) were selected to compose the *SLC25A1*-associated prognostic signature (SPS) gene set, and their LASSO coefficients were listed. According to the expression levels and regression coefficients, the downregulated *BAX, PWP2, SERTAD1, S1PR4, ZFAND1, NUDT13* and *ZNF107* with HR < 1 were considered as tumor suppressors, whereas the *MVD*, *BAD*, *CPNE7*, *CPNE7*, *UTP23* and *ZNF124* upregulated with HR > 1 were regarded as oncogenes (Supplementary Figure S2). We calculated a risk score as follows:

$$\begin{array}{ll} Risk\;Score & =\left(0.51643*MVD\right)+\left(-0.58074*BAD\right)+\left(0.53567*BAX\right)+\left(-0.49648*PWP2\right)\\ & +\left(-0.34582*SERTAD1\right)+\left(0.08634*CPNE7\right)+\left(-0.31038*S1\mathrm{PR}4\right) \end{array}$$

$$+\left(-0.54617*ZFAND1\right)+\left(0.94536*UTP23\right)$$

$$+\left(-0.37781*ZNF124\right)+\left(0.35928*NUDT13\right)+\left(-0.29836*ZNF107\right)$$

In the training set, patients in the Beat AML dataset can be stratified into low- and high-risk subgroups by the cutoff value of the risk scores (Figure 4C, Supplementary Table S5). We run time-dependent ROC analysis to evaluate the prediction efficiency of the SPS. The area under curve (AUC) of 1, 3 and 5 years was .744, .744, .739, respectively (Figure 4D). Moreover, Kaplan-Meier analysis showed that high-risk group of AML patients had a shorter overall survival outcome compared with the low-risk group (*p* < .001) (Figure 4E). This SPS model were further validated using TCGA dataset (Supplementary Table S6) and another validation set TARGET_AML (Supplementary Table S7). In the TCGA dataset, the cutoff value of risk score was -.07 (Figure 4F). The 1-, 3- and 5-year AUC values were .612, .697 and .683, respectively (Figure 4G). The high-risk group had poor OS (n = 100 for low-risk group, n = 56 for high-risk group) (Figure 4H). In the other validation set, similar pattern was observed in TARGET_ AML dataset with -.14 as cutoff value (Supplementary Figure S3A). The 1-, 3- and 5-year AUC values were .642, .541 and .541, respectively (Supplementary Figure S3B). High-risk score patients demonstrated much shorter OS compared to low-risk group in TARGET_ AML dataset (Supplementary Figure S3C). As noted above, these results collectively indicated that SPS had highly accurate prediction value.

### Clinical impact and application of SPS in stratification of AML patients

**FIGURE 5:**
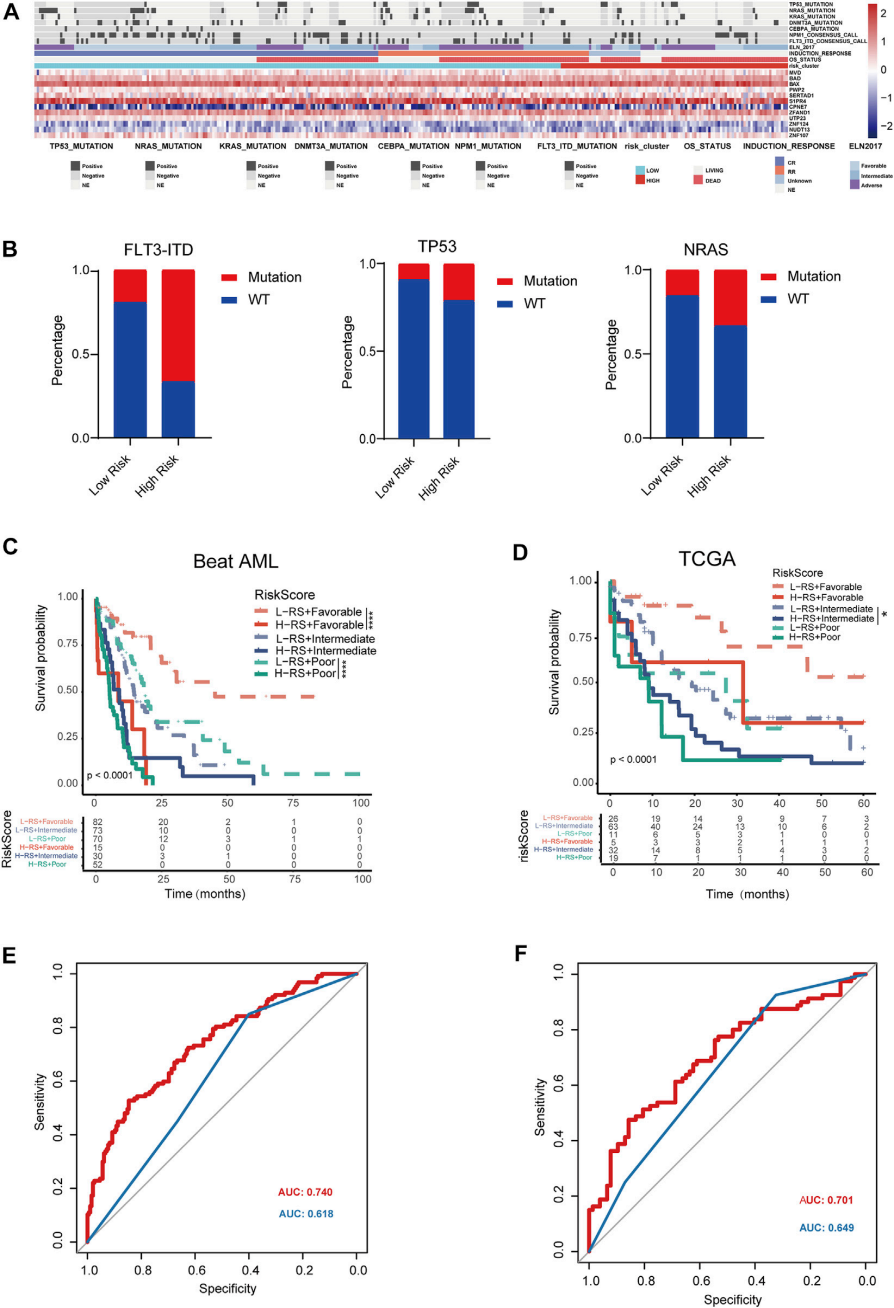
Clinical impact of *SLC25A1*-associated prognostic signature in AML patients **(A)**. Heatmap depicting the distribution of frequently somatic mutation between low and high risk groups **(B)**. Distribution of the percentage of different mutation between high and low-risk groups **(C,D)**. Kaplan-Meier analysis of re-stratification of AML patients by refined SPS-ELN2017 model. Six categories were obtained in the Beat AML dataset and TCGA dataset, respectively **(E,F)**. Predictive value of SPS was compared to conventional ELN2017 risk category for Beat AML dataset and TCGA dataset. Red line and blue line indicated refined SPS-ELN2017 and ELN2017 model, respectively.

To further assess whether our SPS model has an impact and further application on clinical outcomes, we collected clinicopathological data from Beat AML database. We compared the mutation patterns between these two risk score groups (Figure 5A). The analysis of the mutational status of AML SPS-based subgroups revealed that FLT3-ITD, TP53, NRAS mutations were more common in high-risk score subgroup samples. Mutations of these three genes in Beat AML dataset were 66%, 21% and 33%, compared to 20%, 9% and 15% for the low-risk score subgroup (Figure 5B). ELN2017 risk stratification system is widely accepted in a wide range of AML. However, classified patients by ELN2017 still demonstrate substantial prognostic heterogeneity. Therefore, we constructed a refined AML risk stratification model with SPS. By incorporating the SPS, AML patients stratified by the ELN2017 classification could be further divided into six subgroups. Notably, patients with high SPS risk scores had markedly worse outcomes than low SPS risk scores for both the ELN-favorable and ELN-poor groups in the Beat AML dataset (Figure 5C) (*p* < .0001). Moreover, for TCGA dataset, patients with high SPS risk scores had markedly worse outcomes than low SPS risk scores for ELN stratified intermediate group (Figure 5D). We then compared AUC of our refined SPS-ELN2017 prognostic model with ELN2017 in these two different datasets. We find AUC of refined SPS-ELN2017 model (red) were significantly higher than that of ELN 2017 (blue) (Figures 5E, F). Taken together, we found that a refined SPS-ELN2017 model could more accurately predict prognosis of AML patients.

### SPS is an independent prognostic factor

**FIGURE 6:**
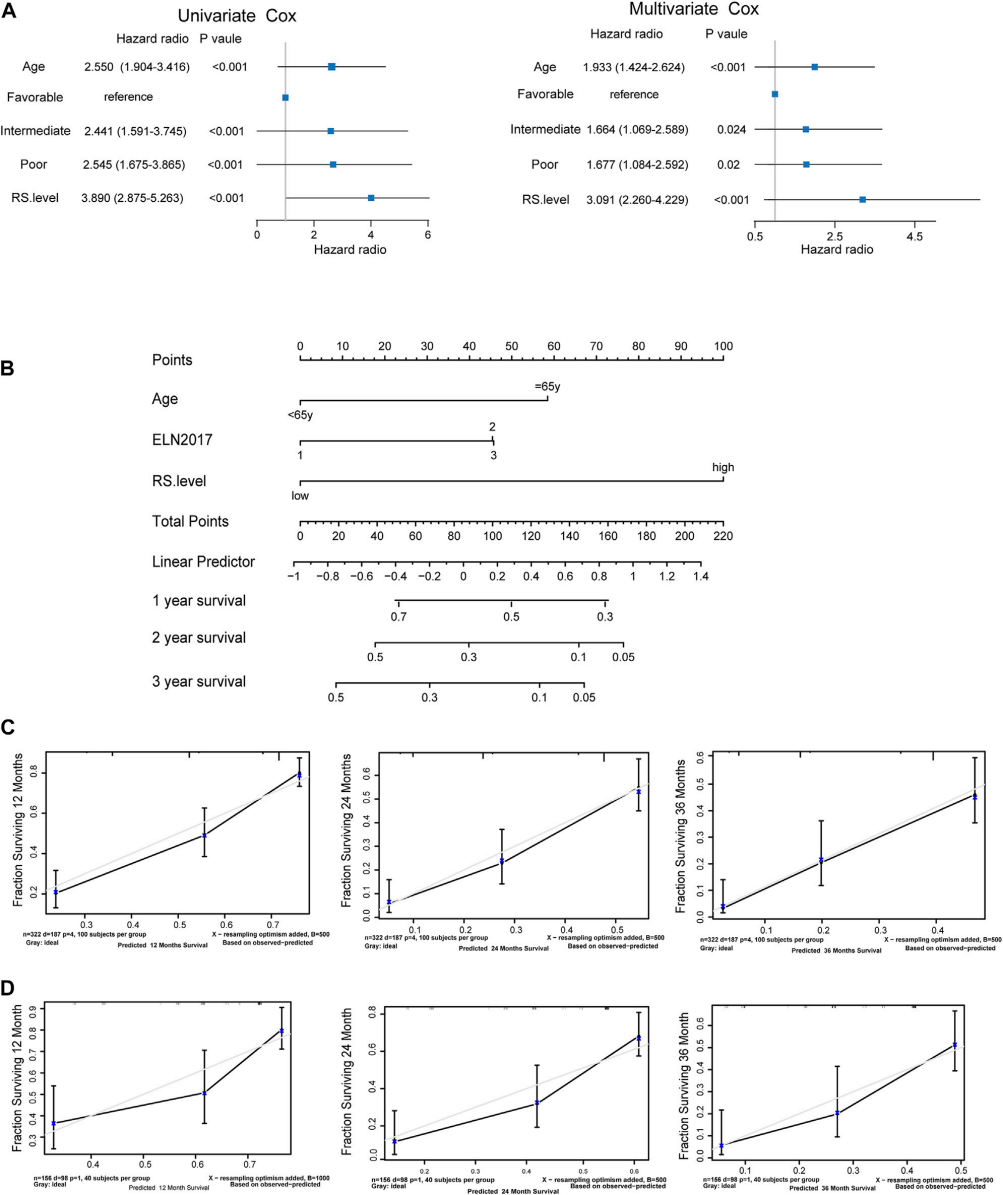
*SLC25A1*-associated prognostic signature is an independent prognostic factor **(A)**. Forrest plots of univariate and multivariable Cox proportional hazards of the *SLC25A1*-associated prognostic signature associated with OS **(B)**. Nomogram for predicting the probability of 1-, 2-, and 3-year OS integrating SPS, ELN2017 and age **(C,D)**. Calibration plots of the nomogram for agreement test between 1-, 2-, and 3-year OS prediction and actual outcome in the Beat AML dataset and TCGA dataset.

To evaluate whether *SLC25A1*-associated prognostic signature was an independent predictive factor of AML, both univariate and multivariate analyses were conducted. In the univariate analysis, age, ELN2017 and risk score were identified to be significantly correlated with prognosis (*p* < .001, *p* < .001, *p* < .001 respectively) in the Beat AML cohort (Figure 6A, left). Notably, the SPS risk score level accounted for adverse overall survival (HR = 3.89, *p* < .001). In addition, multivariate Cox regression also indicated that the SPS risk level was an independent predictor of poor OS (HR, 3.091; 95% CI, 1.424–2.624; *p* < .001) after considering the age (HR, 1.933; 95% CI, 2.26–4.229; *p* < .001), ELN 2017 (HR, 1.667; 95% CI, 1.084–2.592; *p* = .02) (Figure 6A, right). Based on these prognostic factors, a prognostic nomogram was constructed to facilitate clinical prognostic prediction for AML patients with SPS. By assigning a score to each item based on the actual condition, patients could get a total score for predicting their survival rate within 2- and 3-year (Figure 6B). The c-index of the nomogram in the Beat AML dataset was .727. It was higher than that of the 2017ELN risk stratification (.583), indicating that the nomogram embraced better fitting efficacy. The calibration plot for the probability of 1-, 2- and 3-year OS showed a good linear relationship between prediction by the nomogram and actual observations in this dataset (Figure 6C). In addition, the c-index value for the nomogram in the TCGA dataset was .72, which is higher than that of the 2017 ELN risk stratification (.428). Similarly, the prediction by the nomogram and the observed survival rate showed a satisfactory fitting in the TCGA dataset (Figure 6D). Collectively, SPS is an independent prognostic factor and the new nomogram integrating this SPS risk score represents an improved model to predict the outcome of AML.

## Discussion

Acute myeloid leukemia (AML) is the most common type of acute leukemias in adults (Sung et al., 2021). It exhibits high mortality and poor prognosis (Estey, 2020). With the extensive research on the pathogenesis of AML, it has been confirmed that multi-gene biomarkers become a promising prognosis tool for AML (Prada-Arismendy et al., 2017).

Metabolic reprogramming is generally regarded as a hallmark of AML cells (Castro et al., 2019). *SLC25A1* belongs to a large family of nuclear-encoded mitochondrial transporters and exerts the fundamental function of allowing the transit of citric through the impermeable mitochondrial membrane (Catalina-Rodriguez et al., 2012). Citric is the central hub of the mitochondrial metabolism and respiration (Nakhle et al., 2020). For the past few years, the research of *SLC25A1* focused on its functions and mechanisms in solid tumors progression (Im et al., 2014; Fernandez et al., 2018; Yang et al., 2021). In this study, we found that the *SLC25A1* is highly expressed in AML patients and high *SLC25A1* expression is correlated with worse prognosis of AML patients. Next, we found that inhibition of *SLC25A1* reduced the proliferation and promote apoptosis of AML cells. These results indicated that *SLC25A1* may be a factor for predicting the prognosis and a potential therapeutic target of AML patients. Further investigation on the function and mechanism of *SLC25A1* in AML *in vitro* and *in vivo* would provide evidence and new insights on whether it can serve as a therapeutic target to treat AML.

We built a robust prognostic signature based on *SLC25A1* associated genes by LASSO regression and validated it with two independent cohorts. Our SPS-prognostic model might be a promising candidate for predicting prognosis of AML patients. This prognostic model involves twelve SPS genes including mevalonate pyrophosphate decarboxylase (*MVD*), BCL2 Associated Agonist Of Cell Death (*BAD*), BCL2 Associated X (*BAX*), PWP2 Small Subunit Processome Component (*PWP2*), SERTA Domain Containing 1 (*SERTAD1*), Sphingosine-1-Phosphate Receptor 4 (*S1PR4*), Copine 7 (*CPNE7*), Zinc Finger AN1-Type Containing 1 (*ZFAND1*), UTP23 Small Subunit Processome Component (*UTP23*), Zinc Finger Protein 124 (*ZNF124*), Nudix Hydrolase 13 (*NUDT13*), Zinc Finger Protein 107 (*ZNF107*).

These twelve genes were reported to participate in many essential cellular processes, including metabolism, apoptosis, cell cycle and signal transduction. AML cells alters metabolic pathways to meet the increased biosynthetic and energy needs to support enhanced cell growth and survival. The reprogramming of cellular metabolism is a fundamental characteristic of AML. For example, isocitrate dehydrogenase (IDH) is the key enzyme responsible for Tricarboxylic Acid Cycle (TCA) that is one of the most important processes in central metabolism. In AML patients, IDH1/IDH2 somatic mutation frequencies were about 6%–16% and 8%–19%, respectively. With the understanding of biological and clinical properties of mutated isoforms of IDH 1 and IDH2, inhibitors ivosidenib and enasidenib have been developed to teat AML (Platt et al., 2015; Halik et al., 2022). This suggest that targeting aberrant pathways of metabolism might be a promising strategy for antileukemia therapy. Among the twelve genes we identified, *MVD* has an important role in cholesterol biosynthesis (Mazein et al., 2013). *S1PR4* is the Sphingosine-1-phosphate (S1P) receptor that is one of G-protein-coupled receptors (Cartier and Hla, 2019). Cholesterol biosynthesis and sphingolipid metabolism is the central metabolic hub for numerous biological processes in cancer. Therefore, they may be of potential value as new target for AML treatment. Moreover, apoptotic genes are reported to be associated with the development of AML (Roberts et al., 2021). The Bcl-2 family is a key apoptosis regulator in the apoptosis signal transduction pathway, and they participate in a very complex interaction mechanism to regulate apoptosis. *Bcl-2* is a well-recognized target in AML and its inhibitor Venetoclax is already widely used for the treatment of AML in clinic practices (Garciaz et al., 2021). Therefore, it is not surprising that Bcl-2 family members, *BAD* and *BAX* were among this prognostic gene signature (Gayle et al., 2019; Mann et al., 2019). In addition, resistance and relapse of AML are associated with the aberrant regulation of the cell cycle. *SERTAD1* plays an important role in cell cycle progression. *ZFAND1* enables proteasome binding activity and the *SERTAD1* gene is highly expressed in some solid tumors. *ZFAND1* loss causes clearance of stress granules aberrated. stress granules have been proposed to form important signaling hubs (Kedersha et al., 2013),which subsequently affect the survival of tumor cells (Turakhiya et al., 2018). Thus, it may also worth exploration as a potential target for AML treatment. Through other genes within the twelve-gene signature have not been reported previously in AML, these were reported to play important roles in some solid tumors. *PWP2* promoted invasion and migration of Gastric Adenocarcinoma (Zhou et al., 2021). Expression of *UTP23*, *NUDT13* and *CPNE7* are associated with poor prognosis in tumors including ovarian cancer, gastric cancer, oral squamous cell carcinoma (McLennan, 2006; Fu et al., 2019; Ji et al., 2021). Since these twelve gene SPS could improve the accuracy of prognosis analysis, they may represent new vulnerability of AML cells and their function and potential as therapeutic targets are worth further investigation.

Based on this twelve-gene signature, AML patients were divided into the high- and low-score groups accordingly in the training set. We found that the survival of the two risk groups was significantly different. There was an evident prolonged OS time and lower mortality rate of the low-risk group. For ROC analysis of TCGA dataset as validation set, the AUC values of 1-,3- and 5-year survival were .612, .697 and .683, respectively, indicating accuracy of this SPS risk assessment model. Although the AUC value in another validation cohort of TARGET_AML is lower than that in the Beat AML training cohort and the TCGA validation dataset, the prognostic signature still exhibited satisfactory predictive power demonstrated by the Kaplan-Meier survival analysis (*p* < .001). This could be due to TARGET_AML is composed of pediatric AML patients and they might exhibit different metabolic characteristics with adult patients. Nevertheless, the prediction accuracy of our model was adequately enough for valid prediction of independent prognostic factor.

The 2017 European Leukemia Net (ELN 2017) guidelines for the diagnosis and management of AML becomes fundamental guidelines concerning the treatment and estimation of prognosis. In this study, the SPS can further stratify the heterogeneous ELN-favorable, intermediate and poor subgroups. Moreover, we further validated that the SPS was an independent prognostic factor in addition to some clinical factors. In the Beat AML cohort, we found the risk score most significantly affected the survival of AML patients, which can effectively usher prognostic prediction. Calculation of c-index identified that nomogram was higher than .7. As a retrospective study, our study of this new prediction model is still limited. Large-cohort prospective studies collecting transcriptomic data and with extended follow-up of AML patients would further help to evaluate the power of this SPS integrating new model. In summary, due to high degree of accuracy of our combined SPS-ELN2017 model integrating SPS, it is worth consideration for its application to predict prognosis of AML in clinical settings.

## Data Availability

The original contributions presented in the study are included in the article/Supplementary Material, further inquiries can be directed to the corresponding authors.
