# Supplementary material for: SLC25A1-associated prognostic signature predicts poor survival in acute myeloid leukemia patients
Source: Front Genet. 2023 Jan 6;13:1081262. doi: 10.3389/fgene.2022.1081262 (PMC9852877; doi:10.3389/fgene.2022.1081262)
Supplement: Supplementary file 8 [file DataSheet1.docx]

Supplementary Material

**Figure S1**


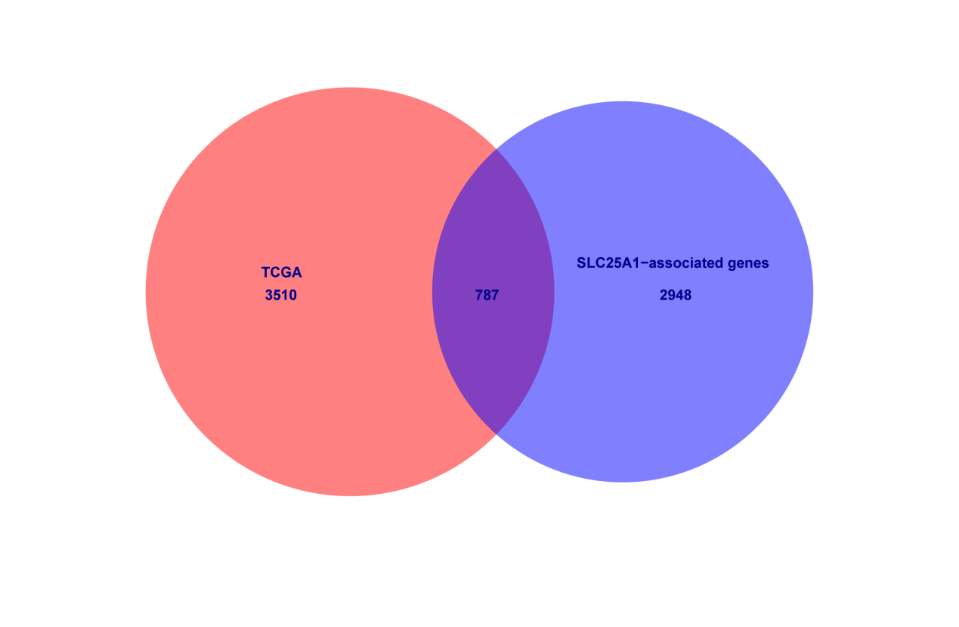


**Figure S1** Venn diagram showing the 787 common genes in 4297 differentially expressed genes in TCGA-GTEx dataset and *SLC25A1*-associated genes

**Figure S2**
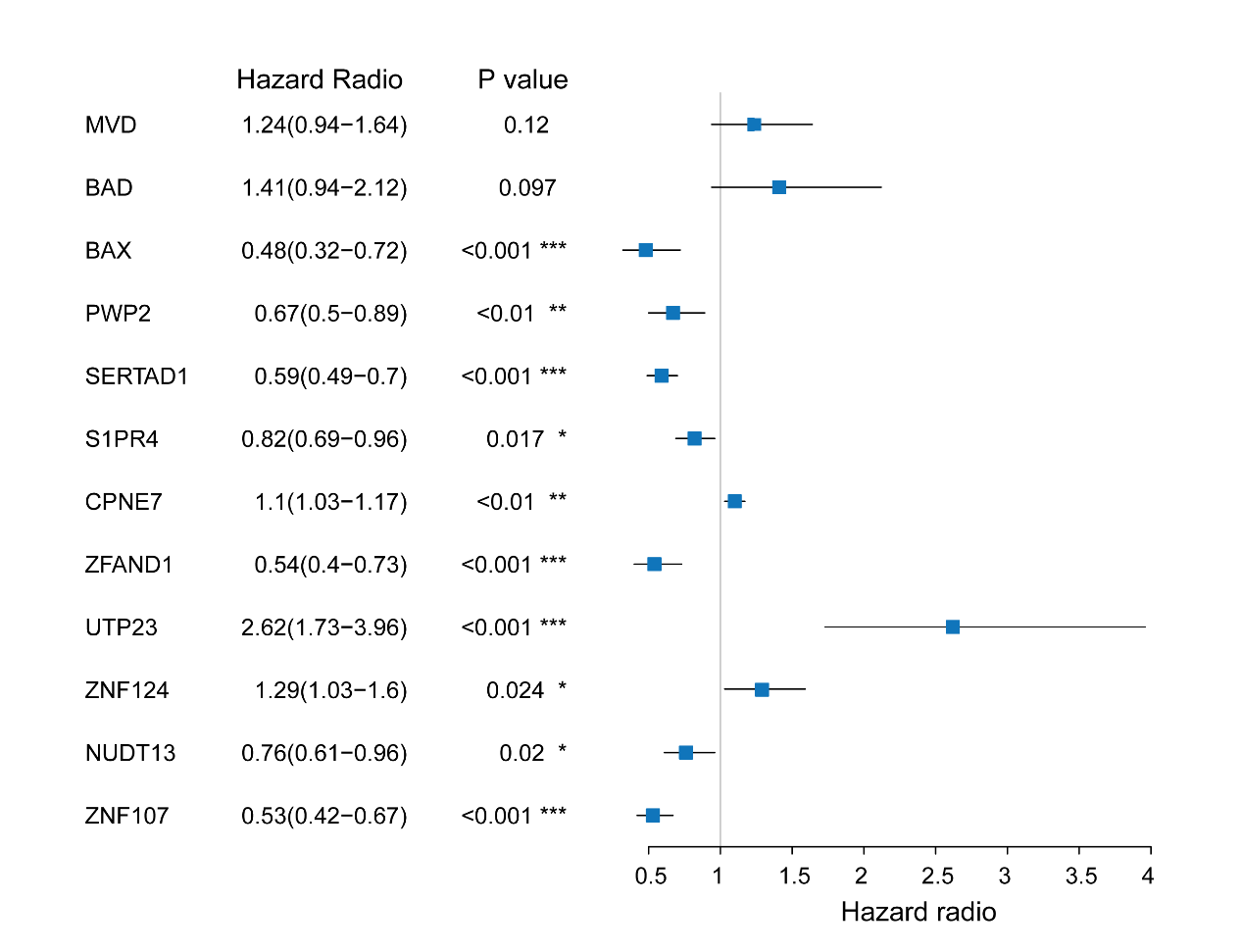


**Figure S2** Multi-variates regression analysis of hazard ratio among 12 prognostic genes were eventually selected after LASSO regression

**Figure S3**


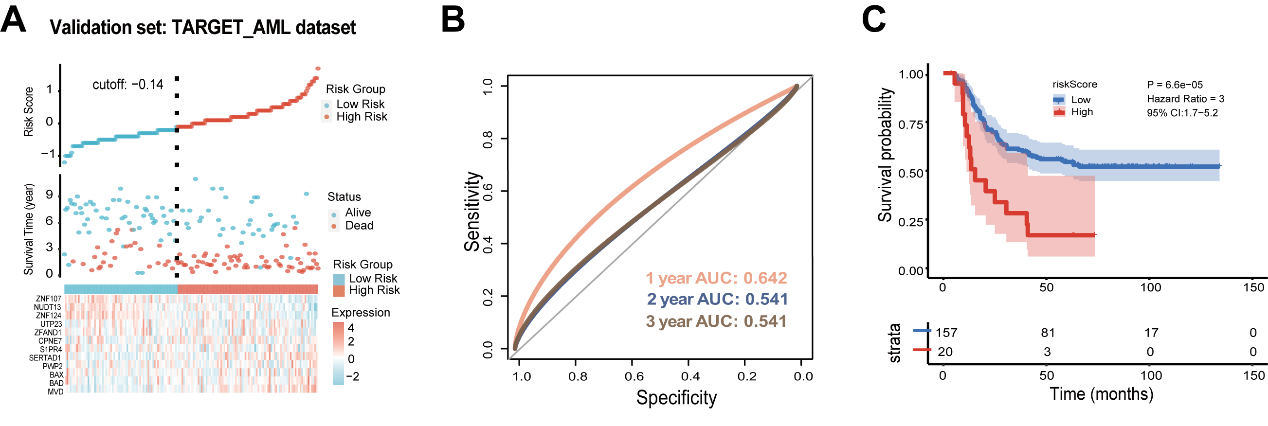
 **Figure S3** Validation of Prognostic Model by TARGET_AML dataset. (**A**)**.** Relationship between survival status/risk and expression heat map of model genes. (**B).** The 1-, 3-, and 5-year ROC curves of risk score. (**C**)**.** Kaplan-Meier curves showing percentages of surviving patients of two different group. *P* value was calculated using the log-rank test.
